# hnRNPC Promotes Malignancy in Pancreatic Cancer through Stabilization of IQGAP3

**DOI:** 10.1155/2022/6319685

**Published:** 2022-03-20

**Authors:** Nannan Yang, Lin Liu, Xiaoyu Liu, Yingjie Chen, Jian Lu, Zhongmin Wang

**Affiliations:** ^1^Department of Radiology, Ruijin Hospital Luwan Branch, Shanghai Jiao Tong University School of Medicine, Shanghai 200020, China; ^2^Department of Interventional Radiology, Ruijin Hospital, Shanghai Jiao Tong University School of Medicine, Shanghai 200025, China

## Abstract

Due to challenges in early-stage detection, aggressive behavior, and poor response to systemic therapy, pancreatic cancer is one of the most fatal cancer types globally. The role of RNA-binding protein (RBP) transcription and translation of cancer cells has been well demonstrated, although their roles in pancreatic cancer is less well understood. In this study, we found that heterogeneous nuclear ribonucleoprotein C (hnRNPC), a RBP, is highly expressed in pancreatic ductal adenocarcinoma (PDAC) tissues and cells. In addition, we discovered that overexpression of hnRNPC in PDAC cells *in vitro* increased cell proliferation, migration, invasion, and metastasis. The presence of hnRNPC promoted tumorigenesis of pancreatic cells in metastatic *in vivo* models, which was also validated. *In silico* analyses revealed that hnRNPC is a strong positive regulator of IQ Motif Containing GTPase Activating Protein 3 (IQGAP3) activity. The experimental confirmation of this association revealed a direct interaction of IQGAP3 and hnRNPC to induce cell growth and invasion in PDAC cells by activating the epithelial-mesenchymal transition. In light of the findings that hnRNPC accelerates PDAC progression by interfering with IQGAP3, it appears that this technique for diagnosis and treatment of PDAC may have promise.

## 1. Introduction

Pancreatic cancer is currently the seventh leading cause of cancer-related death worldwide [1, 2]. An observation made by Wong et al. in both male and female populations of industrialized countries revealed a progressive increase in the prevalence of pancreatic cancer [3], which may be linked to changes in lifestyle factors such as smoking, consumption, eating habits, and obesity [4]. In the United States, pancreatic cancer is predicted to overtake breast cancer as the second highest cause of cancer-related mortality by 2030 [5, 6]. Pancreatic ductal adenocarcinoma (PDAC) is the most common subtype of pancreatic cancer, accounting for around 85% of all cases worldwide. Endocrine tumors are the less common subtype, accounting for fewer than 5% of all cases [7, 8]. Furthermore, with an estimated five-year survival rate of 5% for pancreatic cancer, primary prevention and early detection are the main strategies for improving the overall survival rate [9]. Early identification of pancreatic cancer is now impossible due to the lack of well-established diagnostic tools, and surgery to remove malignant tissue is the sole therapeutic option [4]. As a result, there is a critical need to better understand the molecular etiology of this disease as well as to develop innovative diagnostic and therapy approaches for it.

Currently, serum cancer antigen 19-9 (CA19-9) is the sole FDA-approved marker for diagnostic and routine management of pancreatic cancer [10]. Although CA19-9 has good specificity for distinguishing benign from malignant pancreatic tumors, it has poor sensitivity [11] and can be high in hepatobiliary malignancies and benign biliary obstruction, among other conditions [12]. The most common alteration seen in pancreatic cancer include KRAS protooncogene (KRAS) gene, which regulates the transforming growth factor beta (TGF-*β*), Notch, and Wnt signaling pathways have been extensively studied in pancreatic cancer [13]. Most of these pathways seem to generally modulate cancer through their role in cell apoptosis, proliferation, migration, and chemoresistance [14]. However, these genetic mutations do not allow prognostic assessment, and molecularly targeted therapeutic strategies against KRAS mutations have yet to be proven effective in long-term survival of PDAC patients [14, 15]. MicroRNAs (miRNAs) and RNA-binding proteins (RNABPs) have been identified as potential biomarkers of PDAC. These biomarkers have the potential to aid in the diagnosis of PDAC [16, 17]. Although extensive research into the role of miRNAs in cancer has been conducted, little is known about RBP's role in PDAC [18, 19]. hnRNPC is one such RBP, which is best known for its role in RNA splicing, 3′ end processing, translation, and RNA expression regulation [20–22]. RBPs have recently piqued the interest of researchers in the field of cancer, as high levels of expression have been linked to poor outcomes in patients with hepatocellular carcinoma, glioblastoma, and lung cancer, among other cancers [23–25]. However, relatively less is known about the involvement of hnRNPC in pancreatic cancer.

In this work, we discovered that the hnRNPC was substantially expressed in patients with PDAC. As a result, the goal of this study is to better understand the molecular role of hnRNPC in patients with PDAC, which will allow for the identification of new possible diagnostic and therapy techniques for pancreatic cancer patients.

## 2. Materials and Methods

### 2.1. Clinical Specimens

Tumor tissues and adjacent noncancerous pancreatic tissues were obtained from 14 patients from Ruijin Hospital. Immediately, the tissues were frozen down using snap freeze technique with the aid of liquid nitrogen and stored at -80°C. Patients were all well informed of their participation in the study, and a written consent was obtained from all the participants. Appropriate approval for this study was obtained from the Ethics Committee of Ruijin Hospital.

### 2.2. Cell Culture

Pancreatic cancer cell lines PANC-1, MIApaca-1, SW1990, BxPC-3, and human pancreatic ductal epithelial cells (HPNE) were obtained from ATCC (American Type Culture Collection Center, Virginia, USA). The cells were cultured in RPMI- (Roswell Park Memorial Institute-) 1640 medium (Gibco, Massachusetts, USA) with 10% fetal bovine serum (FBS) and 1% penicillin/streptomycin (Gibco, Thermo Scientific, New York, USA) in the presence of 5% CO_2_ at 37°C. Passaging of the cells were carried out every 2-3 days as the cells reached 70-80% confluency.

### 2.3. Lentiviral Transduction

shRNA specifically targeting the hnRNPC gene, IQ Motif Containing GTPase Activating Protein 3 (IQGAP3) gene, and control scramble shRNA were designed and ligated onto a lentiviral pFH-L plasmid. HEK293-T cells were used for lentiviral packaging, wherein either pFH-L-hnRNPC, pFH-L-IQGAP3, or control shRNA along with helper plasmids pVSVG-I and pCMV*δ*R8.92 (Shanghai Genechem) were transfected using lipofectamine 2000 (Invitrogen) based on manufacturer's instructions. Post 48 h of transfection, the supernatant was collected and passed through a filter (0.45 *μ*m). The collected filtrate was then used to infect the pancreatic cell lines. After 48 h of infection, the cells were washed and collected to perform subsequent Western blotting and PCR experiments.

### 2.4. Cell Growth Assay

Initially, cells were seeded at a density of 5 × 10^3^ cells/96 well. After 24 h of incubation, the proliferation ability of PDAC cells under different treatments was measured using 3-(4,5-dimethylthiazol-2-yl)-2,5-diphenyltetrazolium bromide (MTT). BxPC3 or PANC-1 cells were initially silenced or overexpressed for either hnRNPC or IQGAP3. Further, the cell growth assay was performed at 1, 2, 3, 4, 5, and 6^th^ day. Each day, the cells were incubated with 10 *μ*L MTT (5 mg/mL) in RPMI media (sigma Aldrich, St. Louis, Missouri, USA) for 4 hours. Further, the crystals in the well were dissolved by 100 *μ*L dimethyl sulfoxide (DMSO) for 10 mins (Sigma Aldrich, St. Louis, Mo., USA). Subsequently, the optical density (OD) was measured at 490 nm on the Microplate Reader (Bio-Rad, Hercules, CA, USA).

### 2.5. Colony Formation Assay

Colony formation assay was performed to assess the effect of hnRNPC and IQGAP3 on pancreatic cell lines BxPC3 and PANC-1. After infection with respective lentiviral vectors, cells were seeded at a density of 1 × 10^5^ cells/well in 6-well dish. The cells were further cultured for 10 days at 37°C. Further, the cells were washed with PBS and were fixed using 4% paraformaldehyde (PFA). The cells were then stained using 0.1% crystal violet (sigma, St Louis, MO), and the stained colonies were imaged using microscope. The number of colonies formed were further quantified and analyzed.

### 2.6. Total RNA Extraction and Quantitative Real-Time Polymerase Chain Reaction (qRT-PCR)

Cells and tissues were treated with TRIzol reagent to isolate the total RNA (Invitrogen, USA). 1 *μ*g of the total RNA isolated were reverse transcribed using the reverse transcriptase kit (Invitrogen, Carlsbad, CA, USA). The qRT-PCR was performed using RT-qPCR kit containing SYBR-Green polymerase with the aid of Applied Biosystems Real-Time PCR system (Applied Biosystems). GAPDH was used as an internal control. The primer sequences used were: *hnRNPC* forward, 5′-GCCAGCAACGTTACCAACAA-3′ and reverse, 5′-TGAACAGAGCAGCCCACAAT-3′; and IQGAP3 forward, 5′-GTGCAGCGGATCAACAAAGC-3′ and reverse, 5′-ACGATGCAACAGGGTACACTG-3′; and GAPDH forward, 5′-TGGTATGAGAGCTGGGGAATG-3′ and reverse, 5′-TGGGTGTCGCTGTTGAAGTC-3′. The relative expression was analyzed using 2^−ΔΔCt^.

### 2.7. Transwell Assay

Invasion and migration capacity of the pancreatic cell lines either silenced or overexpressed for hnRNPC or IQGAP3. To assess cell invasion, starvation of cells was achieved for 4 h, trypsinized, and suspended in the serum free medium. The upper chamber's membrane was coated with Matrigel (Corning Life Sciences, NY, USA), and 200 *μ*L of the cell suspension was added to the upper chamber whereas 500 *μ*L of media with 20% serum was supplemented to the lower part of the chamber. Cells were cultured for 24 h, and the cells in the inner part of the membrane were wiped using a cotton swap. The cells were further fixed with 4% PFA for 30 mins and stained with 0.1% crystal violet staining solution. Further, the cells that were positively stained were imaged using an inverted microscope and counted.

### 2.8. CCK-8 Assay

Cell growth was measured in accordance with the instructions for the CCK-8 test kit (Promega, Madison, WI, USA). 48 h after the transfection, the cells were gathered and diluted into 3 × 10^4^ cells/mL. They were then put into 96-well plates, and 10 *μ*L of CCK-8 solution was added to each well. The cells were kept in an incubator with 5% CO_2_ and 37°C for 2 h. With the help of a microplate reader, the OD value at 450 nm was finally tested to detect cell proliferation. The experiment was duplicated three times.

### 2.9. Western Blot Analysis

Cells were treated with RIPA buffer containing 50 mM of Tris, 1% sodium deoxycholate, 150 mM NaCl, 0.1% SDS, 1% Triton X-100 in the presence of phosphatase inhibitor, and PMSF. Using BCA protein quantification assay, we quantified the total protein isolated. Further, protein at a concentration of 20 *μ*g were loaded onto an SDS-PAGE gel and separated. Samples were transferred onto a PVDF membrane and blocked using 5% skim milk. The membranes were then incubated in primary antibodies hnRNPC, IQGAP3, E-cadherin, N-cadherin, Vimentin, and GAPDH. After 24 h, the membranes were incubated with corresponding secondary antibodies conjugated with horse-radish peroxidase. Finally, the membrane was visualized using enhanced chemiluminescence kit (Pierce, Rockford, IL, USA).

### 2.10. Wound Healing Assay

Initially, cells were seeded onto 6 well plates at a density of 5 × 10^4^ cells/well. After 24 h, the cell layer was artificially scratched through the middle of the well using a pipette tip. Then, the wound closure was carefully monitored under a microscope; images were obtained at 0 and 24 h, respectively. The closure of the wound was measured and analyzed with images obtained under same magnification.

### 2.11. RNA Immunoprecipitation (RIP)

Using a Magna RIP RNA-binding protein immunoprecipitation kit (Millipore, Burlington, MA, USA), RIP assay was performed based on the manufacturer's instructions. Initially, lysis of the cells was carried out using RIP lysis buffer. Further, the samples were treated with RIP buffer, which contained magnetic beads conjugated with hnRNPC antibody or control IgG antibody. Further, the collected RNA was quantified using RT-qPCR. The primers used are as previously mentioned.

### 2.12. RNA Pull-Down Assays

RNA was first labeled with biotin using a Biotin-RNA Labeling Mix (Roche, Basel, Switzerland), based on the manufacturer's instructions. Cellular extracts were treated with magnetic beads coated with IQGAP3 to capture RNA-protein complex. Further, the complexed protein (hnRNPC) was separated using Western blotting analysis.

### 2.13. Mouse Xenograft Models

All animal experiments that were performed in this study were evaluated and approved by the Institutional Review Board of Shanghai Jiao Tong University School of Medicine. Five-week-old (*n* = 12) BALB/c nude male mice were obtained from Shanghai Institute of Material Medicine, Chinese Academy of Sciences, and housed under specific pathogen free conditions. PANC-1/BxPC-3 cells (5 × 10^6^ cells) which either overexpressed or lacked hnRNPC were injected subcutaneously onto the right flank of the nude mice. Tumor volumes were carefully assessed every 7 days with a micrometer caliper. The formula used to calculate the tumor volume is tumor volume (mm^3^) = 1/2 *a* × *b*^2^, where *a* indicates the longest latitudinal diameter and *b* indicates the longest transverse diameter. The mice were sacrificed at the end of 5 weeks, and tumors were resected and weighed. Further, the tissue samples were fixed in formalin and processed for immunohistochemistry staining.

### 2.14. Statistical Analyses

The results displayed in this study are presented as mean ± SD. All experiments were repeated for three independent times. Statistical analysis to assess differences between two groups were performed using *t*-test and one-way ANOVA. Kaplan-Meier method were used to plot the overall survival curves. All statistical analysis were performed using SPSS software (v.21, IL, USA). A difference with *P* value < 0.05 was considered as statistically significant.

## 3. Results

### 3.1. hnRNPC Was Highly Expressed in Pancreatic Cancer Tumor Tissues

**Figure 1:**
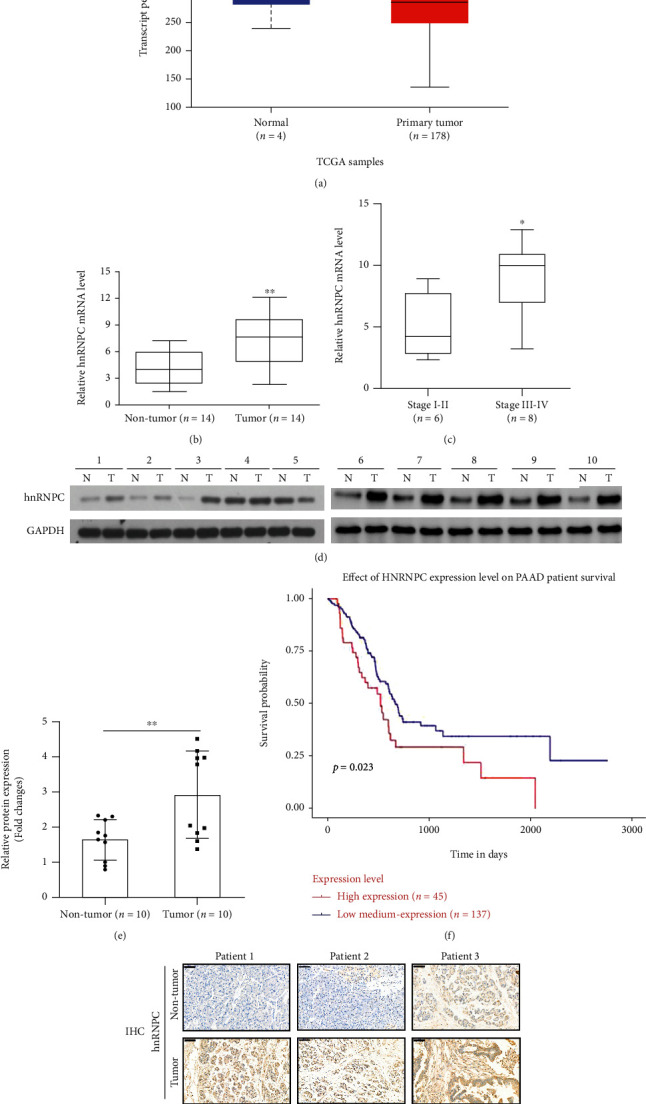
hnRNPC is highly expressed in pancreatic cancer tumor tissues. (a) hnRNPC expression levels in pancreatic adenocarcinoma (PAAD) tissues in TCGA database. (b) Relative mRNA expression levels of *hnRNPC* in 14 paired PDAC tissues and adjacent noncancerous pancreatic tissues, as determined by RT-PCR. (c) Relative mRNA expression of *hnRNPC* in PDAC tissues was stratified by clinical stage, TNM stage I-II (*n* = 6), TNM stage III-IV (*n* = 8). (d, e) Protein expression levels of hnRNPC in patients with PDAC were detected by western blot analysis of the paired PDAC and adjacent noncancerous pancreatic tissues (N: adjacent noncancerous pancreatic tissues; T: tumor tissues, *n* = 10). (f) Overall survival evaluated between pancreatic cancer patients with high and low expression of hnRNPC of pancreatic cancer patients from TCGA. (g) IHC staining of 3 pairs of human PDAC tumor tissues (tumor) and adjacent noncancerous pancreatic tissues (nontumor) for hnRNPC expression. Data were analyzed by a paired Student's *t*-test. Scale bars: 50 *μ*m. ^∗∗^*P* < 0.01.

Initially, we assessed the levels of hnRNPC expression in PDAC tissue samples using data from the Cancer Genome Atlas (TCGA) database. Indeed, we observed that in PDAC tumor samples, hnRNPC transcript levels were slightly higher than that in the normal control samples (Figure 1(a)). Furthermore, we used RT-PCR to examine *hnRNPC* mRNA expression levels in 14 paired PDAC tissues and found that tumor samples had significantly higher levels of hnRNPC than adjacent noncancerous pancreatic tissues; high expression of hnRNPC was positively associated with advanced TNM stage (Figures 1(b) and 1(c)). Additionally, we identified that the levels of hnRNPC protein in PDAC tumor samples were much higher than in adjacent noncancerous pancreatic tissues using Western blot analysis (Figures 1(d) and 1(e)). Using the TCGA database, we identified that overall survival among patients with high hnRNPC was lower compared to the patient with low levels of hnRNPC (Figure 1(f)). Immunostaining of tumor samples confirmed that hnRNPC was highly expressed in PDAC tissue samples compared to the adjacent noncancerous pancreatic tissues (Figure 1(g)).

### 3.2. hnRNPC Promotes PDAC Cell Proliferation, Migration, and Invasion

**Figure 2:**
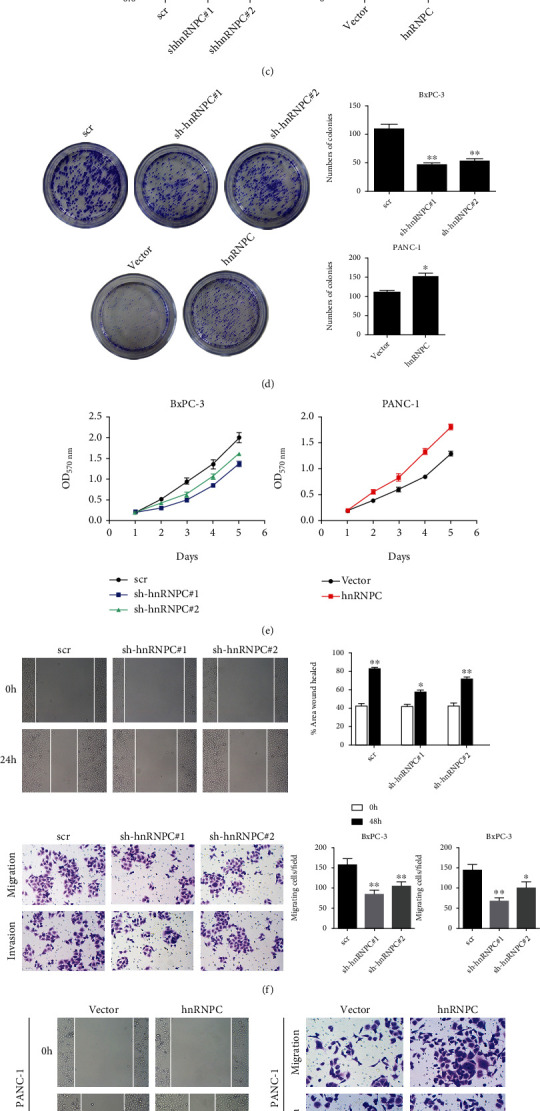
hnRNPC promotes PDAC cell proliferation, migration, and invasion. (a, b) Relative hnRNPC mRNA and protein expression in PANC-1, MIApaca-1, SW1990, and BxPC-3 human pancreatic cells and normal cells (HPNE) by RT-qPCR (*n* = 3; ^∗^*P* < 0.05, ^∗∗^*P* < 0.01, ^∗∗∗^*P* < 0.001 vs. HPNE) (a) and Western blot (b). (c) The expression of hnRNPC protein by qRT-PCR in BxPC-3 cells which were stably knockdown hnRNPC and PANC-1 cells with stable hnRNPC overexpression. (d) Colony formation assays were performed on BxPC-3 cells which were transfected with scr/sh-hnRNPCRNA#1/sh-hnRNPCRNA#2 and on PANC-1 cells transfected with hnRNPC overexpression vector/empty vector (*n* = 3, ^∗^*P* < 0.05, ^∗∗^*P* < 0.01). (e) MTT assay was performed on BxPC-3 cells which were transfected with scr/sh-hnRNPCRNA#1/sh-hnRNPCRNA#2 and on PANC-1 cells transfected with hnRNPC overexpression vector/empty vector to assess cell growth. (f) Cell migration determined by % area wound healed on BxPC-3 cells which were transfected with scr/sh-hnRNPCRNA#1/sh-hnRNPCRNA#2 and cell migration and invasion determined by a Transwell assay on BxPC-3 cells. (g) Cell migration determined by % area wound healed on PANC-1 cells transfected with hnRNPC overexpression vector/empty vector and cell migration and invasion determined by a Transwell assay on PANC-1 cells. Statistical significance was calculated using one-way ANOVA and Student's *t*-tests, respectively. All data shown are the mean ± SD of three independent experiments. ^∗^*P* < 0.05, ^∗∗^*P* < 0.01, ^∗∗∗^*P* < 0.001.

To understand the role of hnRNPC in PDAC, we assessed its expression levels in various human pancreatic tumor cell lines such as PANC-1, MIApaca-1, SW1990, and BxPC-3 with a respective control (HPNE) (Figure 2(a)). Evidently, hnRNPC expression levels were significantly highest in BxPC-3. However, we still could observe high expression of hnRNPC in PANC-1, MIApaca-1, and SW1990, when compared to its control HNPE. Further, we confirmed that hnRNPC protein levels were highest in the BxPC-3 (Figure 2(b)). We consequently knocked down hnRNPC in BxPC-3 and overexpressed hnRNPC in PANC-1 to elucidate its role in PDAC proliferation, migration, and invasion. We confirmed that the amount of hnRNPC was lower in sh-hnRNPC-BxPC-3 cells (interfere with hnRNPC expression in BxPC-3 cells) and higher in hnRNPC-PANC-1 cells (overexpression of hnRNPC in PANC-1 cells) when compared to their respective controls using qRT-PCR (Figure 2(c)). BxPC-3 cells displayed a high colony forming capacity in colony formation experiments but knocking down hnRNPC in these cells drastically reduced their colony forming capacity (Figure 2(d)). Alternatively, PANC-1 cells had very low colony forming capacity and upregulation of hnRNPC significantly increased the colony forming units (Figure 2(d)). Initially, we noticed that BxPC-3 cells proliferated rapidly, and knocking down hnRNPC reduced the proliferation potential of BxPC-3 cells (Figure 2(e)). The PANC-1 cells had lower proliferation capacity and overexpression of hnRNPC increased its proliferation rate (Figure 2(e)). We performed scratch assays to assess PDAC cell's wound healing/migration capacity. BxPC-3 cells had significantly higher wound healing capacity, which was decreased in hnRNPC knockdown cells (Figure 2(f)). To assess hnRNPC's role on migration and invasion, we performed Transwell assays and observed that BxPC-3 cells had high migration and invasion capacity, whereas a knockdown of hnRNPC significantly decreased its migration and invasion capacity (Figure 2(f)). Subsequently, PANC-1 cells had lower wound healing capacity, which could be increased when hnRNPC was upregulated (Figure 2(g)). PANC-1 displayed low levels of migration and invasion, which could be significantly increased by hnRNPC overexpression (Figure 2(g)).

### 3.3. hnRNPC Interacts with IQGAP3 and Promotes Its Expression

**Figure 3:**
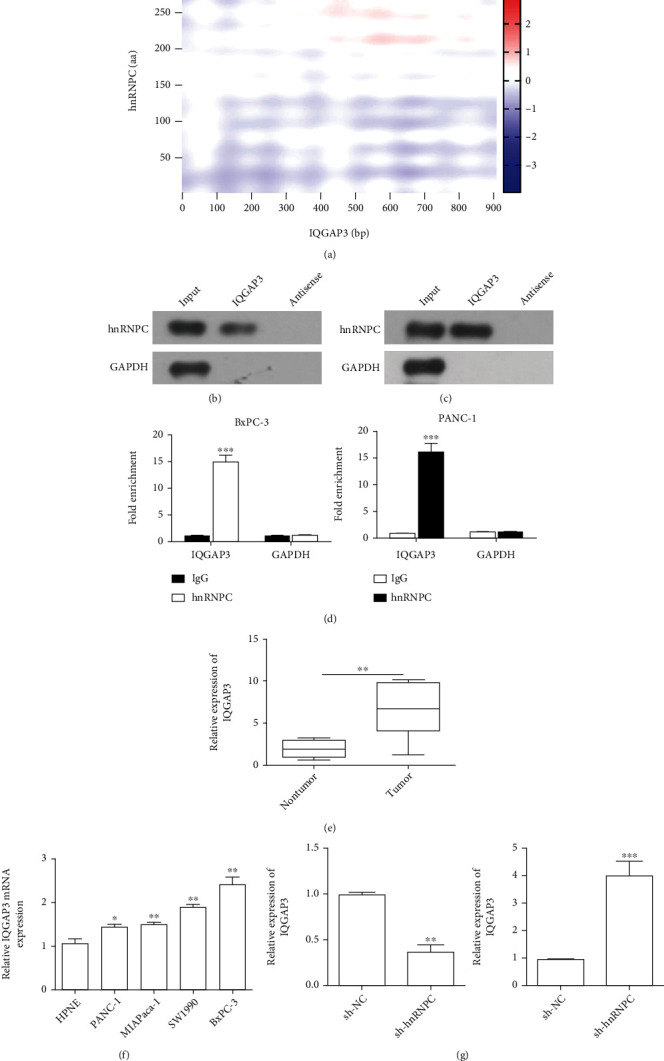
hnRNPC interacts with IQGAP3 and promotes its expression. (a) catRAPID fragment-based prediction analysis of interaction between IQGAP3 and hnRNPC identified a strong interaction with high confidence levels (interaction propensity = 41 and discriminative power = 89%). (b, c) Western blotting analysis of the specific association between hnRNPC and IQGAP3 from RNA pull downs, incubated with lysed cell BxPC-3 (b) and cell PANC-1 (c) supernatant. GAPDH was used as a control. (d) Fold enrichment of IQGAP3 expression in RIP assays performed with hnRNPC coated beads. (e) Relative expression of hnRNPC in PDAC tissues (*n* = 6) and adjacent healthy tissues (*n* = 6) determined by qRT-PCR. (f) Relative expression of IQGAP3 determined in PDAC cell lines PANC-1, MIApaca-1, SW1990, and BxPC-3, compared to HPNE using qRT-PCR and normalized to GAPDH expression. (g) Expression levels of IQGAP3 determined in BxPC-3 and PANC-1 cells expressing sh-hnRNPC or hnRNPC overexpression, respectively. All data shown are the mean ± SD of three independent experiments. Statistical significance was calculated using Student's *t*-test or one-way ANOVA. ^∗∗^*P* < 0.01, ^∗∗∗^*P* < 0.001.

Using predication analysis, we further identified that hnRNPC interacts with IQ Motif Containing GTPase Activating Protein 3 (IQGAP3). catRAPID fragment-based prediction analysis identified strong interaction between IQGAP3 and hnRNPC with an interaction propensity of 41% and discriminative power of 89% at high confidence levels (Figure 3(a)). We further performed RNA pull-down assay, wherein BxPC-3 cell lysates (Figure 3(b)) or PANC-1 cell supernatant (Figure 3(c)) were incubated with IQGAP3 synthetic RNA beads, and the bound protein was observed through Western blotting. With the presence of a strong band against hnRNPC antibody, these results clearly indicate the strong interaction between hnRNPC protein and IQGAP3 RNA. Further, to confirm the endogenous interactions between hnRNPC and IQGAP3, RNA immunoprecipitation assays were performed (Figure 3(d)). Here, we used hnRNPC-coated beads, which allowed quantification of the bound IQGAP3 using qRT-PCR. Interestingly, we found that IQGAP3 was significantly enriched in the hnRNPC fractions from both BxPC-3 and PANC-1 cell lines, when compared to the IgG fraction (Figure 3(d)). Next, we checked the expression levels of IQGAP3 using qRT-PCR (Figure 3(e)) and found significantly high levels of IQGAP3 in the tumor samples compared to the adjacent nontumor samples. The assessment of PDAC cell lines using qRT-PCR showed significantly high expression levels of IQGAP3 in PANC1, MIApaca-1, SW1990, and BxPC-3 cell lines, when compared to control HPNE cells. The highest expression of IQGAP3 was observed in BxPC-3 and the lowest in PANC1 cell line (Figure 3(f)). To further confirm the role of hnRNPC in the regulation of IQGAP3, we performed qRT-PCR on samples obtained from cells with either hnRNPC overexpression plasmid or sh-hnRNPC (Figure 3(g)). These results indicated that when hnRNPC was silenced, a significant decrease in IQGAP3 RNA levels could be observed. Alternatively, overexpression of hnRNPC significantly increased IQGAP3 levels. These results clearly confirmed that indeed hnRNPC binds and positively regulates the IQGAP3 levels.

### 3.4. hnRNPC Upregulated IQGAP3 Expression and Promoted Tumor Growth in a Mouse Xenograft Model

**Figure 4:**
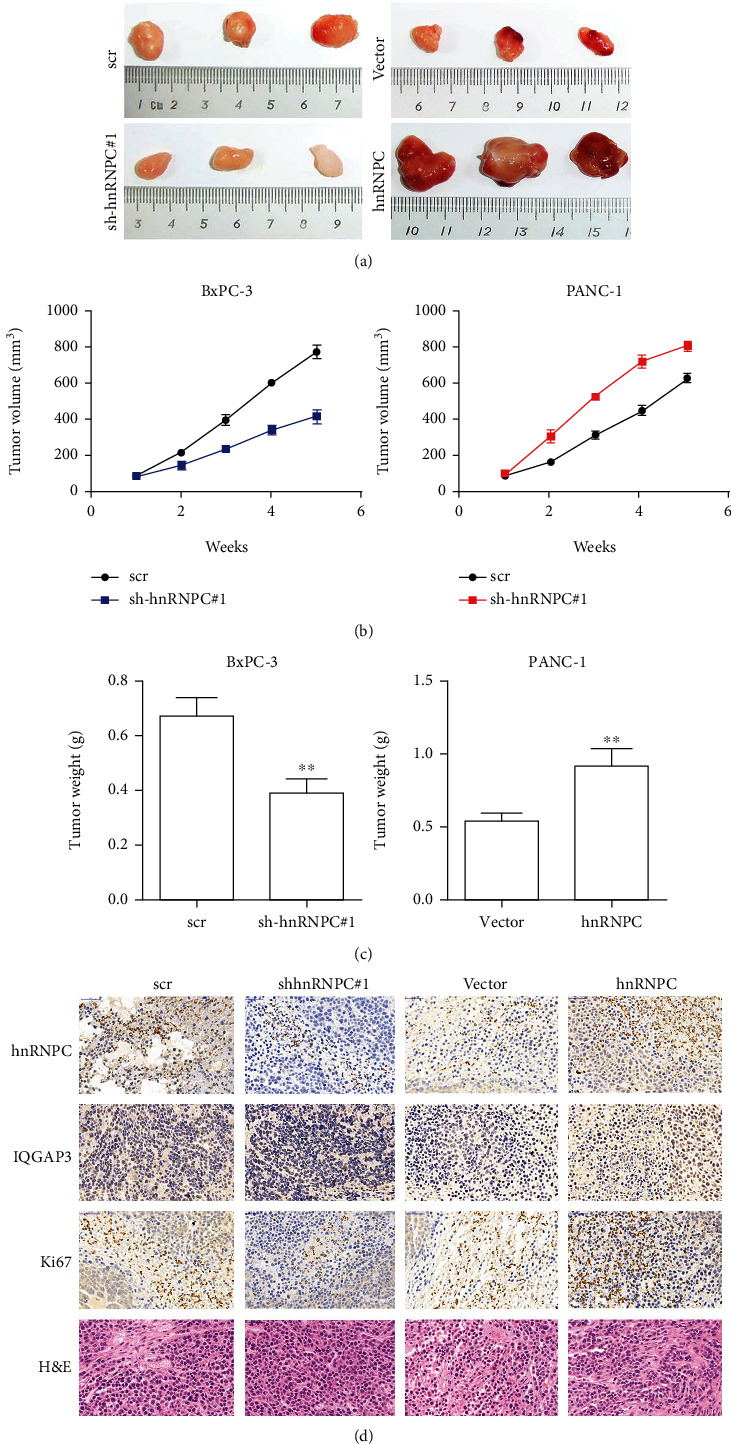
hnRNPC upregulated IQGAP3 expression and promoted tumor growth in a mouse xenograft model. (a–c) BxPC-3 cells with hnRNPC stable knockdown and PANC-1 cells with stable hnRNPC overexpression were injected subcutaneously into nude mice (1∗10^6^ cells per mouse, five mice per group). Representative images of tumors are presented; tumor growth curves (b) and tumor weights (c) are shown. (d) hnRNPC and IQGAP3 expression in four groups was detected by IHC. H&E staining and Ki-67 immunohistochemical staining of tumor sections. 400x magnification. All data shown are the mean ± SD of three independent experiments. Statistical significance was calculated using Student's *t*-test or one-way ANOVA. ^∗^*P* < 0.05, ^∗∗^*P* < 0.01, ^∗∗∗^*P* < 0.001.

To further strengthen our *in vitro* results, we assessed the role of hnRNPC in mouse xenograft models. Initially, BxPC-3 or PANC-1 with either a stable knockdown or overexpression of hnRNPC were subcutaneously injected into nude mice (1 × 10^6^ cells per mouse, five mice per group). Mice were sacrificed after 6 weeks of study, and the tumors were removed. It was obvious from this results that hnRNPC knockdown cells reduced tumor weight and size in mice injected. On the other hand, tumor weight and size increased dramatically in mice injected with cells overexpressing hnRNPC (Figures 4(a) and 4(c)). The tumor's volume was also measured throughout the course of 6 weeks. We found that tumor volume increased in mice overexpressing hnRNPC, whereas tumor volume decreased in mice injected with hnRNPC knockdown cells, relative to their significant controls (Figure 4(b)). Using immunohistochemistry, we further assessed the tumor samples for expression of hnRNPC, IQGAP3, and Ki-67. Samples from the sh-hnRNPC group displayed decreased expression of hnRNPC, IQGAP3, and Ki-67. However, mice samples from hnRNPC overexpression group, showed clear increase in hnRNPC, IQGAPs, and Ki-67 expression, compared to its respective control (Figure 4(d)). Additionally, using H&E staining, we also observed the extent of tumorigenesis in these tissues (Figure 4(d)). The above-mentioned results further strengthened our *in vitro* observations, which indicated that hnRNPC plays a key role in upregulation of IQGAP3 and progression of tumorigenesis.

### 3.5. hnRNPC Promotes PDAC Cell Proliferation, Migration, Invasion, and EMT through IQGAP3

**Figure 5:**
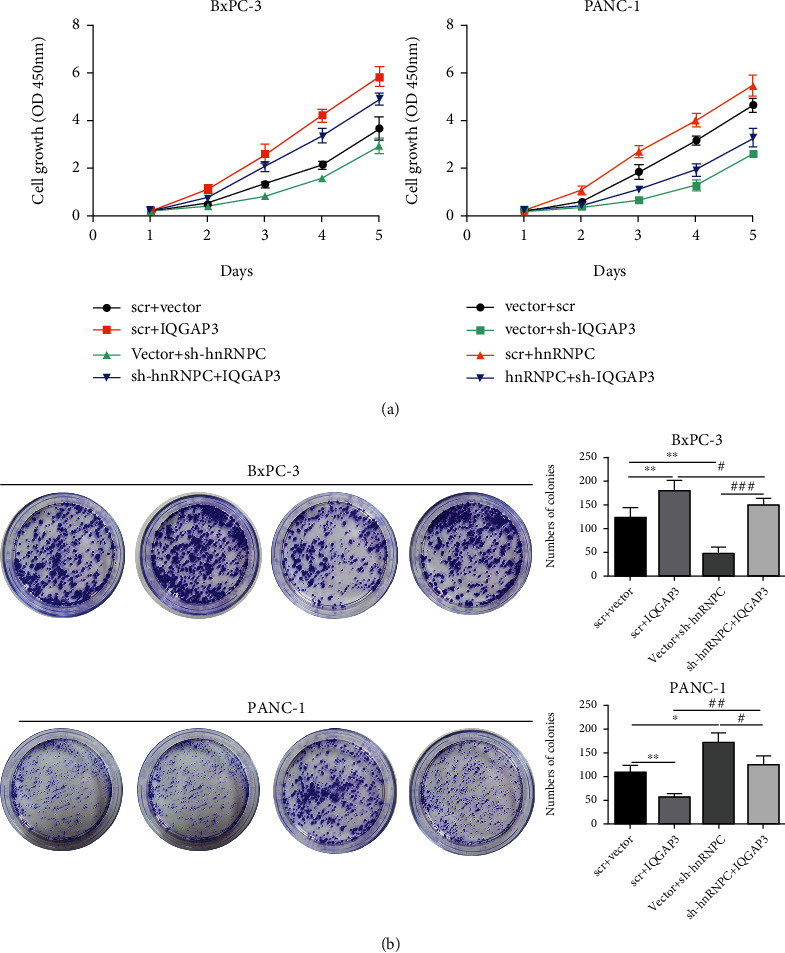
hnRNPC promotes PDAC cell proliferation through IQGAP3. (a, b) Cell proliferation and colony formation were determined in BxPC-3 and PANC-1 cells transfected with different shRNA control (scr) or sh-hnRNPC, sh-IQGAP3 along with IQGAP3 or hnRNPC overexpression, respectively. All data shown are the mean ± SD of three independent experiments. Statistical significance was calculated using Student's *t*-test or one-way ANOVA. ^∗^*P* < 0.05, ^∗∗^*P* < 0.01 vs. sh-NC+NC, ^#^*P* < 0.05, ^##^*P* < 0.01 vs. sh-hnRNPC + IQGAP3 or sh-IQGAP3+hnRNPC.

**Figure 6:**
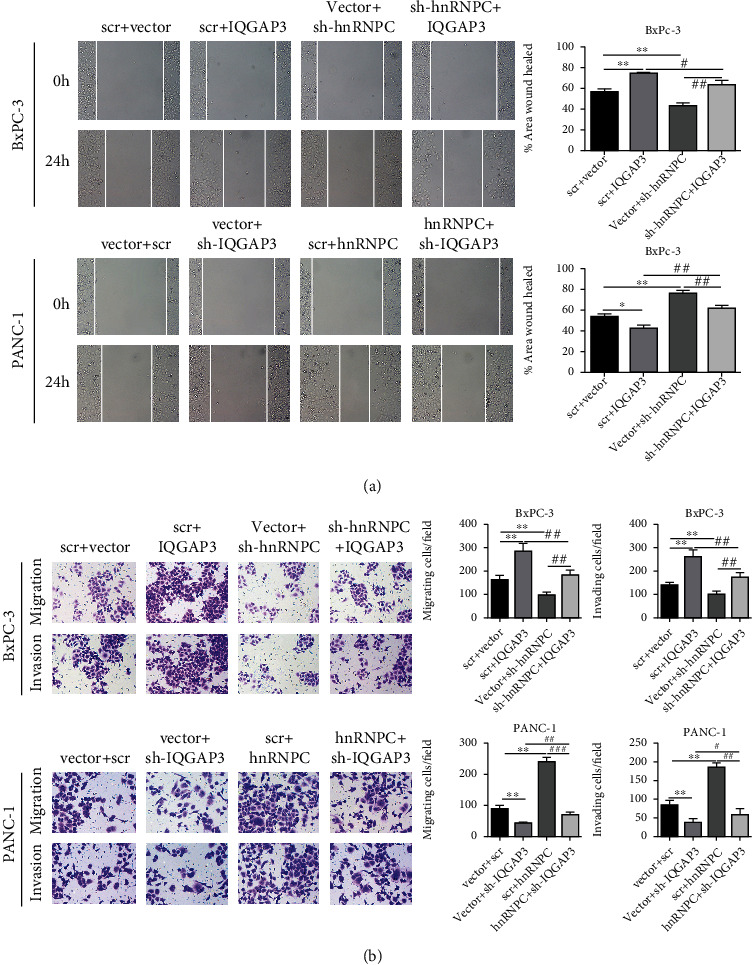
hnRNPC promotes PDAC cell migration and invasion through IQGAP3. (a) Cell migration as determined by percent area wound healed in BxPC-3 and PANC-1 cells transfected with the respective scr or overexpression plasmids. (b) Cell migration and invasion were determined in BxPC-3 and PANC-1 cells transfected with the respective scr or overexpression plasmids by Transwell assays. All data shown are the mean ± SD of three independent experiments. Statistical significance was calculated using Student's *t*-test or one-way ANOVA. ^∗^*P* < 0.05, ^∗∗^*P* < 0.01 vs. sh-NC+NC, ^#^*P* < 0.05, ^##^*P* < 0.01 vs. sh-hnRNPC + IQGAP3 or sh-IQGAP3+hnRNPC.

We performed experiments on BxPC-3 or PANC-1 cells that were either overexpressing or missing IQGAP3 to see if hnRNPC influenced PDAC carcinogenesis through IQGAP3. First, CCK-8 assay was performed to detect cell proliferation and discovered that BxPC-3 cells overexpressing IQGAP3 showed a consistent increase in proliferation when compared to their respective control cells. The alternate silencing of IQGAP3 in PANC-1 resulted in a decrease in proliferation, demonstrating that IQGAP3 plays a critical role in the progression of PDAC. Simultaneously, the proliferation of cells was reduced by silencing the hnRNPC gene. Cells recovered and multiplied more rapidly when IQGAP3 was overexpressed at the same time. The cell proliferation was also unable to be restored when both IQGAP3 and hnRNPC were silenced at the same time. These findings clearly demonstrated that IQGAP3 rather than hnRNPC has a direct impact on PDAC progression (Figure 5(a)). Such findings were also confirmed in colony formation experiments, where IQGAP3 overexpression resulted in enhanced colony number formation while silencing of hnRNPC at the same time only resulted in a slight decrease in the number of colony forming units. However, silencing of IQGAP3 caused a significant decrease in colony formation, and overexpression of hnRNPC could only slightly improve the colony numbers (Figure 5(b)). Next, we also observed similar results in scratch wound healing, migration, and invasion assays (Figures 6(a) and 6(b)). That the hnRNPC-IQGAP3 pathway is an important regulator of PDAC tumor growth has been validated by these findings.

**Figure 7:**
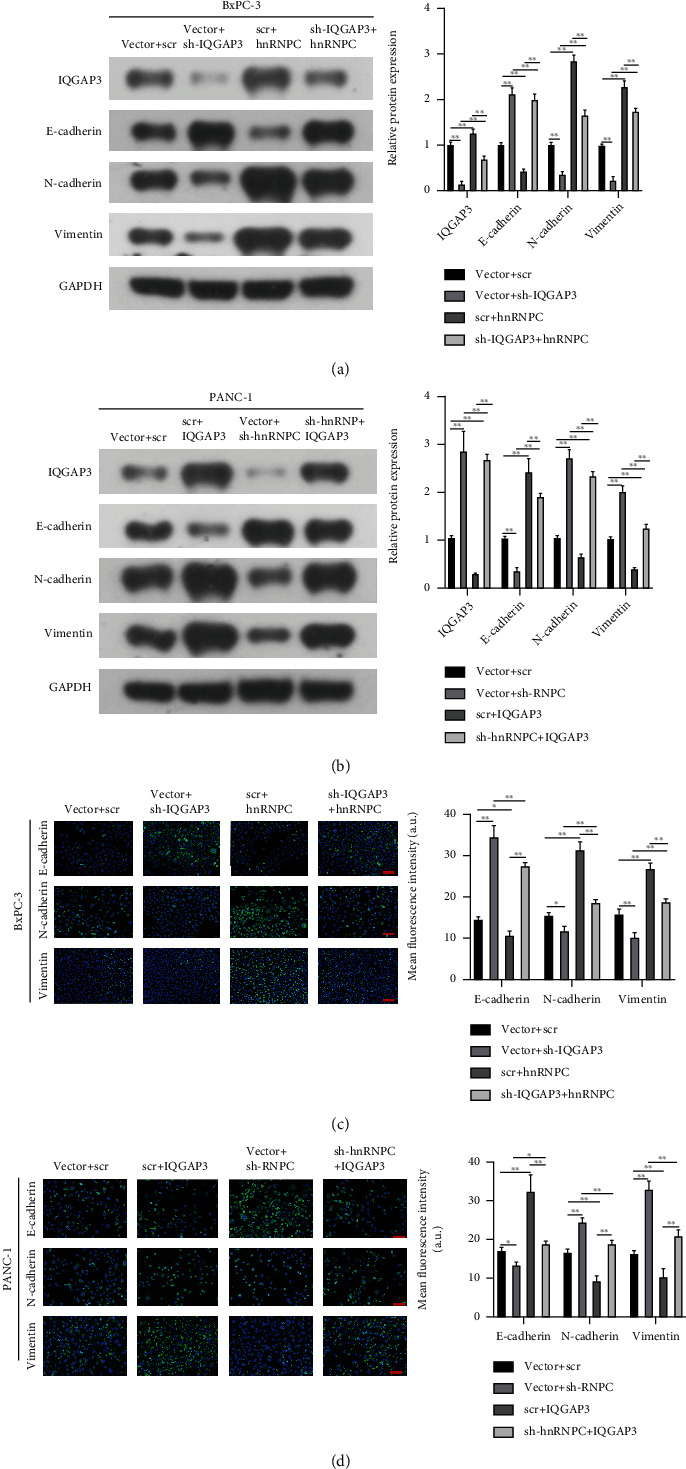
hnRNPC promoted PDAC cell EMT through IQGAP3. (a, b) Western blot analysis of EMT marker proteins (E-cadherin, N-cadherin, and vimentin) and IQGAP3 in BxPC-3 and PANC-1 cells transfected with the respective shRNA or overexpression plasmids and normalized by GAPDH. (c, d) Representative immunofluorescence images of EMT protein (E-cadherin, N-cadherin, and vimentin) expression levels in BxPC-3 and PANC-1 cells transfected with the respective shRNA or overexpression plasmids, bar = 50 *μ*m. All data shown are the mean ± SD of three independent experiments, ^∗^*P* < 0.05, ^∗∗^*P* < 0.01.

We further assessed the role of hnRNPC and IQGAP3 in tumor progression by tracing the expression of key epithelial-mesenchymal transition (EMT) markers such as E-cadherin, N-cadherin, and vimentin. Using Western blotting and immunostaining (Figure 7). We assessed the alterations in BxPC-3 and PANC-1 cell lines which had been transfected with either shRNA or overexpression plasmids. In BxPC-3 cells, silencing of IQGAP3 decreased N-cadherin and vimentin, while increasing E-cadherin levels. Alternatively, overexpression of hnRNPC increased N-cadherin and vimentin levels, while decreasing E-cadherin levels. However, simultaneously silencing IQGAP3 and overexpressing hnRNPC clearly decreased N-cadherin and vimentin levels, while increasing E-cadherin levels (Figures 7(a) and 7(c)). Additionally, overexpression of IQGAP3 in PANC-1 cells resulted in a decrease in E-cadherin levels while simultaneously raising N-cadherin and vimentin levels. Silencing of hnRNPC increased E-cadherin while decreasing N-cadherin and vimentin levels. E-cadherin levels were marginally enhanced when hnRNPC was silenced, and IQGAP3 was overexpressed; however, the levels of N-cadherin and vimentin were decreased. IQGAP3 is critical for EMT transition, and hnRNPC regulates IQGAP3 to govern this transition, according to these studies.

## 4. Discussion

Despite the limited amount of information on the role of RBPs in cancer, it is clear that RBPs play an important role in tumorigenesis. According to previous studies, one group of RBPs known as the insulin-like growth factor 2 mRNA-binding protein family (IGF2BPs) has been found to be significantly elevated in PDAC [26–28]. In particular, three members of this family, IGF2BP1, 2, and 3, have received extensive research attention for their protumorigenic features, which include enhanced proliferation, migration, and metastasis. IGF2BP1 was discovered to influence pancreatic cancer carcinogenesis by modifying the Akt signaling pathway, IGF2B2 by directly binding to and stabilizing GLUT1 mRNA, and IGF2B3 through regulating mRNA-miRNA interaction [26–28]. These studies do establish the importance of RBPs in tumorigenesis. Remarkably, we observed that hnRNPC, an RBP, is highly expressed in PDAC and is associated with poor prognosis in pancreatic cancer (Figure 1(f)). hnRNPC has long been known to play important roles in transcription, translation, 3′ end splicing, and RNA stability [20–22]. hnRNPC has also been shown to be a modulator of many important oncogenic genes, including breast cancer susceptibility gene (BRCA), metastasis associated in lung denocarcinoma transcript 1 (MALAT1), and c-Myc [29–31]. Downregulation of hnRNPC, in particular, appears to significantly reduce protumorigenic properties of cancer cell lines via regulation of the interferon cascade pathway in breast cancer [32]. Downregulation of hnRNPC in glioblastoma inhibited its binding to miRNA-21, which suppressed the Akt pathway via upregulation the expression of programmed cell death 4 (PDCD4). Interestingly, this reduced glioblastoma cell's migratory and invasive capacity, inducing antitumorigenesis in these cells [25]. Similarly, in this study, we demonstrated that hnRNPC increased tumorigenic properties such as proliferation, migration, invasion, and colony formation in pancreatic cell lines PANC-1 and BxPC-3 (Figures 1 and 2). Further, using in silico analysis, we identified that hnRNPC binds to and stabilizes IQGAP3 mRNA (Figure 3(a)). With additional silencing and overexpression experiments, we were able to observe that IQGAP3 does, in fact, directly regulate oncogenesis via EMT modulation (Figures 4–6). Further, using *in vivo* models, we were able to confirm that hnRNPC increased the volume, weight, and growth of xenograft tumors by binding and stabilizing IQGAP3 (Figures 5 and 6).

IQGAP3 is a GTPase activating protein that has been linked to fundamental cellular processes such as cell proliferation, adhesion, cytoskeletal remodelling, and growth factor regulation [33]. There has recently been an increase in the number of studies on its role in tumorigenesis of the lungs, colorectum, liver, gastric, and breast [33–36]. Surprisingly, majority of these studies suggest that IQGAP3 modulates tumorigenesis by regulating the EMT and thus controlling tumor metastasis. In lung cancer, this is accomplished through IQGAP3's regulation of EGFR-ERK signaling [34], whereas in hepatocellular carcinoma, this is accomplished by promoting TGF-*β*, which is a key EMT regulator [33]. Growing evidence has indicated that EMT is a vital process in metastasis and reoccurrence of many cancers. Hence, in this study, we further explored the influence of IQGAP3 in EMT of PDAC cell lines. We observed that overexpression of IQGAP3 decreased E-cadherin levels while increasing N-cadherin and vimentin levels in PDAC cells, which is a classic indicator of the transition from epithelial to mesenchymal state (Figure 7). This is the first work to show that hnRNPC binds and stabilizes IQGAP3, allowing it to play an essential role in modulating PDAC proliferation, migration, invasion, and EMT, even though IQGAP3 was previously discovered to be increased in pancreatic cancer cell lines [37].

We believe there is a strong need to understand the molecular etiology of pancreatic cancer and to identify key biomarkers for both prognostic determination and the development of novel treatment strategies as there are few to no available diagnostic and treatment strategies. In this study, we identified that hnRNPC regulates pancreatic cancer tumorigenesis by binding and stabilizing IQGAP3, and thus, this study allows identification of novel diagnostic markers and potential targets to develop treatment strategies for pancreatic cancer.

## Data Availability

The datasets used and/or analyzed during the current study are available from the corresponding author on reasonable request.
